# PRDX1 is essential for the viability and maintenance of reactive oxygen species in chicken DT40

**DOI:** 10.1186/s41021-021-00211-4

**Published:** 2021-08-05

**Authors:** Takahito Moriwaki, Akari Yoshimura, Yuki Tamari, Hiroyuki Sasanuma, Shunichi Takeda, Masayuki Seki, Keizo Tano

**Affiliations:** 1 Department of Molecular and Genetic Medicine, Kawasaki Medical School, 577, Matsushima, Kurashiki-city, Okayama 701-0192 Japan; 2 Division of Biochemistry, Faculty of Pharmaceutical Sciences, Tohoku Medical and Pharmaceutical University, 4-4-1 Komatsushima, Aoba-ku, Sendai, Miyagi 981-8558 Japan; 3 Department of Radiology, Kyoto Prefectural University of Medicine, Kajii-cho, Kawaramachi-Hirokoji,Kamigyo-ku, Kyoto, 602-8566 Japan; 4 Department of Radiation Genetics, Graduate School of Medicine, Faculty of Medicine, Kyoto University, Yoshida-Konoe-cho, Sakyo-ku, Kyoto, 606-8501 Japan; 5 Department of Biological Sciences, Graduate School of Science, Osaka Prefecture University, 1-1 Gakuen-cho, Naka-ku, Sakai, Osaka, 599-8531 Japan

## Abstract

**Background:**

Peroxiredoxin 1 (PRDX1) is a member of a ubiquitous family of thiol peroxidases that catalyze the reduction of peroxides, including hydrogen peroxide. It functions as an antioxidant enzyme, similar to catalase and glutathione peroxidase. PRDX1 was recently shown act as a sensor of reactive oxygen species (ROS) and play a role in ROS-dependent intracellular signaling pathways. To investigate its physiological functions, PRDX1 was conditionally disrupted in chicken DT40 cells in the present study.

**Results:**

The depletion of PRDX1 resulted in cell death with increased levels of intracellular ROS. PRDX1-depleted cells did not show the accumulation of chromosomal breaks or sister chromatid exchange (SCE). These results suggest that cell death in PRDX1-depleted cells was not due to DNA damage. 2-Mercaptoethanol protected against cell death in PRDX1-depleted cells and also suppressed elevations in ROS.

**Conclusions:**

PRDX1 is essential in chicken DT40 cells and plays an important role in maintaining intracellular ROS homeostasis (or in the fine-tuning of cellular ROS levels). Cells deficient in PRDX1 may be used as an endogenously deregulated ROS model to elucidate the physiological roles of ROS in maintaining proper cell growth.

## Introduction

Peroxiredoxins (PRDXs) are highly ubiquitous cysteine-dependent peroxidases that remove peroxides, including hydrogen peroxide, organic hydroperoxides, and peroxynitrite [1–4]. In most vertebrates, the PRDX family comprises 6 isoforms (PRDX1–6), which are classified into two subfamilies, 1-Cys and 2-Cys, based on the number of conserved active cysteine residues [5, 6]. PRDX1 is a member of the 2-Cys PRDX1 subfamily and the oxidation of two cysteines is required to activate its enzymatic activity [7]. One cysteine residue at the N terminus of PRDX1 is oxidized to detoxify peroxides through intermolecular disulfide formation with the other cysteine residue at the C terminus [7, 8]. PRDX1 localizes to the cytosol and is abundant in cultured mammalian cells [9]. In addition to its antioxidant enzymatic activity, various functions have been reported for PRDX1. It has been shown to play roles in tumor suppression [10–13], apoptosis [14–16], and molecular chaperoning [17, 18]. A previous study demonstrated that *PRDX1* knockout mice were viable and fertile, but developed severe hemolytic anemia and several malignant cancers, which shortened their life span [10]. Another *PRDX1* knockout mouse study showed elevated nuclear reactive oxygen species (ROS) levels in primary tissues isolated from these mice with increased DNA damage and tumor susceptibility [12]. However, the physiological role of PRDX1 in the oxidization-reduction balance remains unclear.

We have been investigating the physiological roles of ROS by targeting genes involved in ROS scavenging using a reverse genetics strategy in chicken DT40 cell lines derived from an avian leukosis virus (ALV)-induced bursal lymphoma. Superoxide dismutase 1 (SOD1) and 2 (SOD2) were systematically disrupted to generate conditional mutant cells in which the human *SOD1* or *SOD2* transgene was expressed under the control of a tetracycline-inducible promoter [19–21]. These cell lines enabled us to eliminate the exogenous effects of ROS and characterize phenotypes caused by endogenously disturbed ROS levels. As a part of this strategy, we generated PRDX1 conditional mutant cells in which the expression of *Gallus Gallus domesticus PRDX1*(*ggdPRDX1*) may be turned off by a treatment with doxycycline (DOX) and examined cellular responses immediately after the depletion of PRDX1. The results obtained demonstrated that the depletion of PRDX1 resulted in cell death with increases in total ROS levels and genome integrity was maintained. The lethality of PRDX1-deficient cells was completely suppressed by the addition of a reductant, 2-mercaptoethanol. Based on the present results, the maintenance of finely tuned cellular ROS levels, particularly hydrogen peroxide, by PRDX1 appears to be a crucial factor for normal cell metabolism.

## Materials and methods

### Cell culture

Cells were cultured in RPMI 1640 supplemented with 10% fetal bovine serum and 1% chicken serum at 39 °C under 5% CO_2_ without a reductant for a normal culture [22]. To investigate the effects of various antioxidants on cell viability, cells were cultured in the presence or absence of DOX for the indicated periods and then treated with antioxidants, 200 μM ascorbic acid phosphate magnesium salt (Wako, Osaka, Japan), 5 mM N-acetyl-L-cysteine (NAC, Wako, Osaka, Japan), 250 μM 1,2-dihydroxy-3,5-benzenedisulfonic acid (Tiron, Sigma-Aldrich), or 50 μM 2-mercaptoethanol (Et-SH, Sigma-Aldrich). Growth curves were generated as previously described [19–21].

### Cell lines used in this study

The *SOD1*^−/−^ + *hSOD1* cell line was generated as described [20]. Briefly, since the knockout of the *SOD1* gene in DT40 is lethal, a conditional *SOD1* knockout with a human *SOD1* gene under the control of a tetracycline-inducible promoter was generated (the *SOD1*^−/−^ + *hSOD1* cell line). A conditional *PRDX1*-knockout cell line with a *ggdPRDX1* gene was generated in this study.

### Plasmid construction

DNA containing exons 2–4 of *PRDX1* was obtained by PCR from DT40 genomic DNA using the Easy-DNA Kit (Invitrogen, Carlsbad, California, USA) and Ex-Taq polymerase (Takara Bio Inc., Otsu, Shiga, Japan). The chicken targeting constructs for *PRDX1*, *PRDX1*-blasticidin and *PRDX1*-ecogpt, were generated by replacing exons 2–4 with the blasticidin or ecogpt selection marker cassette. To construct an expression plasmid carrying *ggdPRDX1* cDNA (*ggdPRDX1cDNA*) with the tet-off promoter, *ggdPRDX1* cDNA was obtained by reverse transcription-PCR (RT-PCR) using mRNA from DT40 cells using SuperScript III Reverse Transcriptase (Invitrogen) and Flag-tagged *PRDX1* cDNA inserted into the pUHG 10–3 vector.

### Measurement of intracellular ROS

Intracellular ROS levels were measured using 2′,7′-dichlorodihydrofluorescein diacetate (DCFH-DA; Molecular Probes, USA). Intracellular peroxide-dependent oxidation converts DCFH-DA to the fluorescent compound 2′,7′-dichlorofluorescein (DCF), as previously described [23]. After washing antioxidant treated or un-treated cells with PBS, cells were incubated with DCFA-DA (20 μM) at 37 °C for 30 min, harvested, and resuspended in 50 mM HEPES buffer (5 mM HEPES, pH 7.4, 5 mM KCl, 140 mM NaCl, 2 mM CaCl_2_, 1 mM MgCl_2_, and 10 mM glucose). Fluorescence intensity was assessed using FACScan (Becton Dickinson, Franklin Lakes, NJ) with excitation at 485 nm and emission at 530 nm.

### Measurement of intracellular superoxide levels

Intracellular superoxide levels were detected using BES-So-AM (Wako Pure Chemical Industries Ltd.), a highly specific fluorescent probe for superoxide [24]. Briefly, cells were treated with 5 μM BES-So-AM for 20 min. After washing twice with PBS, cells were suspended in PBS, and fluorescent intensity was measured using FACScan.

### Measurement of sister chromatid exchange (SCE)

An analysis of sister chromatid exchange was performed as previously described [19, 20]. Briefly, cells were cultured for two rounds of the cell cycle in medium containing 10 μM BrdU and 0.1 μg/ml colcemid for 2 h. Cells were harvested and treated with 75 mM KCl at room temperature for 18 min and fixed with methanol-acetic acid (3:1) for 30 min. The cell suspension was then dropped onto wet glass slides. After air drying, cells on the slides were incubated with Hoechst 33258 in phosphate buffer (pH 6.8) and rinsed with MacIlavine solution. After cells had been exposed to black light (λ = 352 nm) for 20 min, they were stained with 3% Giemsa solution for 20 min.

### Analysis of chromosomal aberrations

An analysis of chromosomal aberrations was performed as described previously [22]. Briefly, cells were treated for 2.5 h with medium containing 0.1 μg/ml colcemid (Gibco). Harvested cells were incubated in 1 ml of 75 mM KCl at room temperature for 15 min and fixed in 5 ml of a freshly prepared 3:1 mixture of methanol-acetic acid. The cell suspension was dropped onto a slide, and after air drying, cells were stained with 5% Giemsa solution (pH 6.4, Nacalai Tesque, Japan) for 8 min. Data are shown as macro chromosomal aberrations per 50 metaphase spreads.

### Statistical analysis

Three independent experiments were performed for each data set unless stated otherwise. The results obtained are expressed as the mean ± SD unless stated otherwise. The significance of differences was examined using the Student’s *t*-test, and *p* values of < 0.05 were considered to be significant. A multiple-comparison one-way ANOVA was performed using Tukey’s test.

## Results

### Generation of conditional PRDX1 knockout cells

**Fig. 1:**
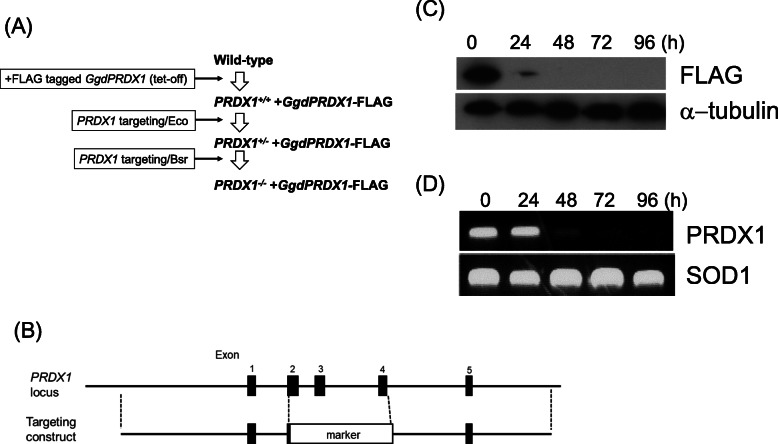
Generation of *PRDX1* conditional mutant cells. **A** Schematic representation of the *PRDX1* conditional mutant of chicken DT40 cells. *ggdPRDX1* cDNA was obtained by reverse transcription-PCR using mRNA from DT40 cells. DT40 wild-type cells were transfected with a plasmid expressing FLAG-tagged *ggdPRDX1 ggdPRDX1* cDNA by the tet-off promoter followed by disruption of genomic *PRDX1* gene. **B** Schematic representation of the disruption constructs. *PRDX1* genomic DNA was cloned into a plasmid, and exons 2, 3, and 4 of *PRDX1* were replaced with a selection marker. See Materials and Methods in details. **C** The Dox-dependent disappearance of chicken PRDX1 as confirmed by Western blot analysis with α-tubulin as the loading control. **D** The Dox-dependent disappearance of chicken PRDX1cDNA as confirmed by a RT-PCR analysis with SOD1 as the control

To investigate the physiological roles of PRDX1 without exogenous oxidative stress, we generated conditional *prdx1* cells in which the *ggdPRDX1* transgene was expressed under the control of the tetracycline-inducible promoter. The expression of the *PRDX1* transgene was switched off by the addition of DOX, which resulted in the complete depletion of exogenous PRDX1 proteins. The strategy of generating conditional PRDX1-deficient cells is shown in Fig. 1A. DT40 wild-type cells were transfected with a plasmid expressing FLAG-tagged *ggdPRDX1* cDNA driven by the tet-off promoter. *PRDX1* genes were then disrupted as shown in Fig. 1B. Disruption was confirmed by Southern blotting (data not shown). The suppressed expression of the *PRDX1* gene and the depletion of the PRDX1 protein were confirmed by RT-PCR and Western blotting, respectively. The transcript and protein levels of PRDX1 both disappeared 96 h after the addition of DOX (Fig. 1C and D). These cells enabled us to characterize cellular responses caused by the depletion of PRDX1 without exogenous oxidative stress.

### PRDX1 is essential for normal cell growth and et-SH protected against cell death in PRDX1-depleted cells

**Fig. 2:**
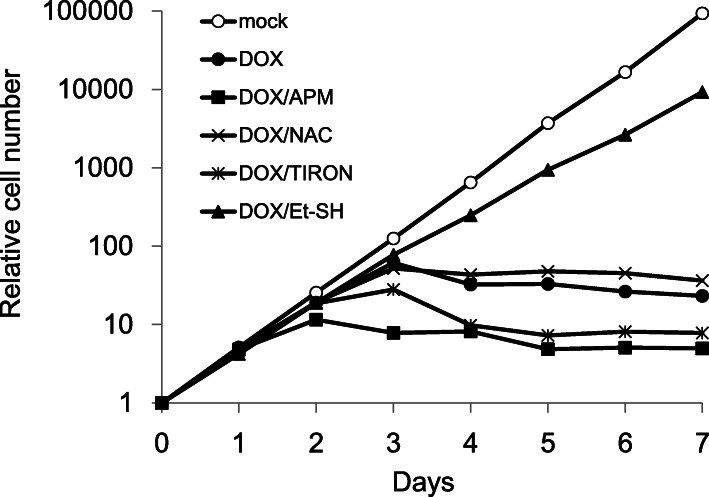
Effects of antioxidants on growth profiles of PRDX1-depleted cells. Cells were incubated with or without 1 μg/ml Dox in the absence or presence of various antioxidants

To elucidate the function of PRDX1, we characterized cellular phenotypes that emerged after the depletion of PRDX1. In contrast to previous finding showing that cells derived from the PRDX1 knockout mouse were viable [10, 11], PRDX1-depleted cells ceased exponential growth on the 3rd day and cell death was observed concomitant with the suppressed expression of PRDX1 after the addition of DOX (Fig. 2).

We previously reported that growth perturbations in SOD1- and SOD2-depleted cells were completely restored by the antioxidant ascorbic acid using L-ascorbic acid phosphate magnesium salt (APM) (19,21). To examine the impact of various antioxidants on cell death in PRDX1-depleted cells, we compared the growth properties of cells depleted of PRDX1 in the presence of the antioxidant APM, the non-toxic dietary glutathione precursor NAC, the widely used mitochondrially targeted antioxidant 4,5-dihydroxy-1,3-benzene disulfonic acid (Tiron), and the strong reducing agent Et-SH, which reduces disulfide bonds with antioxidant capacity by scavenging hydroxyl radicals. Only Et-SH protected against cell death in PRDX1-depleted cells (Fig. 2).

### Et-SH suppresses overall increases in oxidative stress in PRDX1-depleted cells

**Fig. 3:**
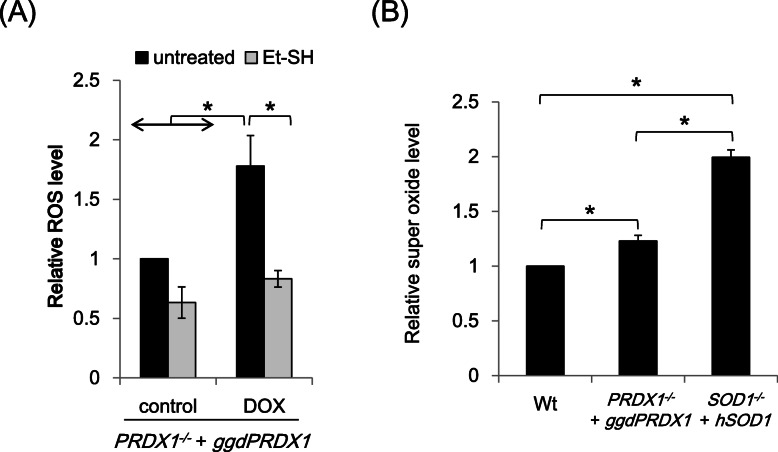
Suppression of elevated cellular oxidative stress in PRDX1-depleted cells by 2-mercaptoethanol. **A** Total cellular oxidative stress was detected by the oxidation of the fluorescent probe DCFH. **B** Cytoplasmic superoxide levels in PRDX1-depleted cells. Superoxide levels were measured with BES-So-AM. Cells were treated with or without 1 μg/ml Dox in the absence or presence of 50 mM 2-mercaptoethanol (Et-SH) for 96 h. SOD1-depleted cells were treated with 1 μg/ml Dox for 96 h used as a positive control in the cytoplasmic superoxide analysis (**B**). Data from flow cytometric analyses are shown as mean fluorescence intensity (MFI). MFI without the Dox treatment was used as the standard (arbitrary unit = 1). All data represent the mean ± standard deviation from three independent experiments. Asterisks (*) indicate *p* < 0.05 by a multiple-comparison one-way ANOVA (Tukey’s test)

PRDXs constitute a family of thiol-dependent peroxidases that scavenge several peroxides, particularly hydrogen peroxide [1–4]. The inefficient scavenging of peroxides in PRDX1-depleted cells may result in an overall increase in oxidative stress in cells. To examine total ROS levels of in cells, we stained PRDX-depleted cells with DCFH. DCFH is a cell-permeable fluorescein dye that reacts with a broad spectrum of cellular ROS [23]. Total ROS levels were elevated in PRDX1-depleted cells (Fig. 3A). Importantly, Et-SH restored ROS to a physiological level in PRDX1-depleted cells (Fig. 3A).

Hydrogen peroxide is a secondary ROS that is generated from superoxide by SOD1 or SOD2. It is readily converted to reactive hydroxyl radicals [25, 26]. Therefore, we investigated whether an increased amount of hydrogen peroxide is derived from superoxide. To compare cytoplasmic superoxide levels in wild-type cells and PRDX1-depleted cells, we stained cells with the highly specific fluorescent probe to superoxide, BES-So-AM [24]. As shown in Fig. 3B, superoxide levels were slightly higher in PRDX1-depleted cells than in wild-type cells. However, this slight increase in superoxide in PRDX1-depleted cells did not account for the total increase in oxidative stress detected by DCFH staining (Fig. 3A). We speculate that increased ROS levels in PRDX1-depleted cells were mainly due to the accumulation of peroxides.

### Depletion of PRDX1 does not affect genome integrity

**Fig. 4:**
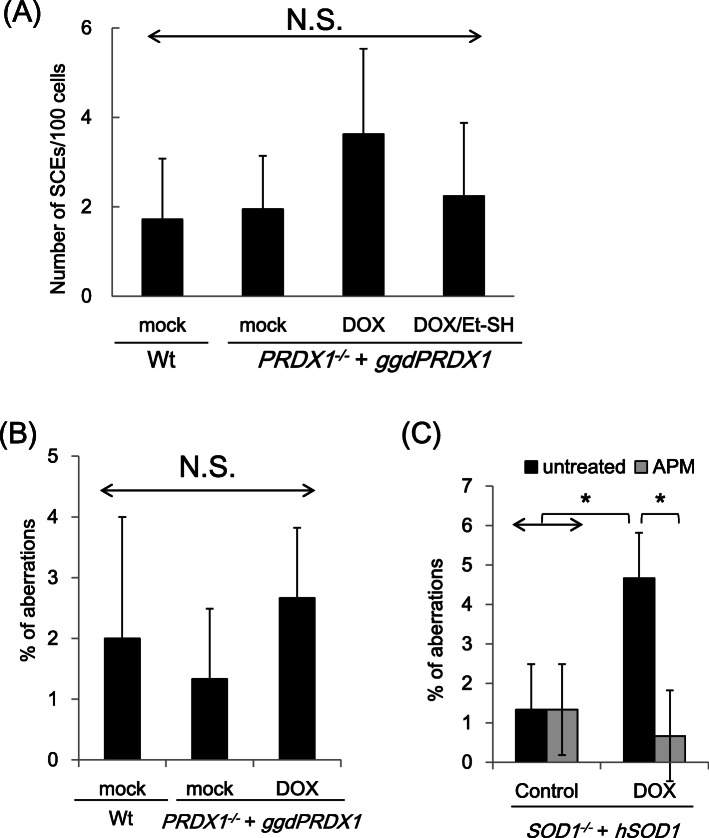
PRDX1 depletion does not induce chromosomal instability. **A** Sister chromatid exchange (SCE). PRDX1-depleted cells were cultured in 1 μg/ml Dox for 96 h and SCE in macrochromosomes was assessed. Numbers represent the means and standard deviations of scores from 100 metaphase cells. **B** Chromosome aberrations. A chromosome analysis was performed with PRDX1-depleted cells. **C** Chromosome aberrations. A chromosome analysis of was performed with SOD1-depleated cells. SOD1-depleated cells were cultured with or without 1 μg/ml Dox for 96 h and /or ascorbic acid (APM) 200 μM for 96 h. Increase of chromosome aberrations in SOD1-depleated cells was suppressed by APM. The number of chromosome aberrations per 50 metaphase nuclei from the indicated cells was counted. All data represent the mean ± standard deviation from three independent experiments. Asterisks (*) indicate *p* < 0.05 by a multiple-comparison one-way ANOVA (Tukey’s test), and N.S. indicates not significant (*p* ≥ 0.05)

Elevated ROS levels induce damage to cellular macromolecules, including DNA and RNA, and genomic instability, resulting in cell death. We previously reported that a chromosome integrity indicator, the frequency of SCE with increases in oxidative stress, was approximately four-fold higher in SOD1-depleted cells than in wild-type cells [20]. To assess the impact of the depletion of PRDX1 on genomic integrity, we examined the frequency of SCE following its depletion. No significant differences were observed in the frequency of SCE between PRDX1-depleted and wild-type cells (Fig. 4A). We also measured the number of chromosomal breaks, which is direct evidence of chromosomal abnormalities in cells. While chromosomal breakage was more prominent in SOD1-depleted cells, it was not induced by the depletion of PRDX1 (Fig. 4B and C). APM suppressed increased of chromosomal breakages (Fig. 4C) as well as growth perturbations in SOD1-depleted cells [21]. These results indicate that cell death in PRDX1-depleted cells was not attributed to the accumulation of chromosomal damage caused by elevated ROS levels.

## Discussion

PRDXs are conserved hydrogen peroxide-scavenging enzymes that also function as key regulators of multiple pathways responding to hydrogen peroxide, including molecular chaperone activity, under elevated oxidative stress as well as the transduction of redox signals [27, 28]. PRDX1 has recently emerged as a modulator of aging-dependent nutrient signaling [29]. In the present study, we demonstrated that PRDX1 was essential for normal cell growth in chicken DT40 cells. Cell death in PRDX1-deficient cells was not associated with chromosomal damage despite the accumulation of cellular ROS (due to large standard deviations in Fig. 4, we are not able to eliminate a possibility formally that PRDX1-depetion causes chromosomal damage at a low level). Hydrogen peroxide acts as a signaling molecule that regulates biological processes due to its potential to induce structural alterations in proteins by preferentially oxidizing cysteine residues. The oxidation of specific amino acids in proteins that are essential for their roles in physiological processes alters their functions and activities [30]. For example, critical cysteine residues in disulfate bound in ataxia-telangiectasia mutated (ATM) can be directly oxidized and activated by a ROS-dependent, but DNA damage-independent manner [31]. We speculate that the attenuation of the fine balance between ROS production and elimination, which is maintained in a PRDX1-dependent manner, is indispensable for normal cell proliferation.

We previous reported that mitochondrial SOD2-depleted cells showed growth perturbations without chromosomal aberrations [19]. We also reported that increased ROS in concomitant with growth perturbation in SOD2-depleted cells were rescued by APM, but not by Et-SH [21]. These results suggested that the perturbation of ROS levels in mitochondria induces cell death without generating chromosomal damage, and APM might scavenge different species of ROS from Et-SH. However, we cannot eliminate the possibility that ROS-induced DNA damage in mitochondrial DNA causes cell death in PRDX1-depleted cells. Among the 6 PRDX family genes reported in vertebrates, PRDX5, which contains the mitochondrial targeting sequence, is absent in the aves genome [32, 33]. Aves PRDX1 localizes to the cytosol but may have an unknown role in the maintenance of mitochondrial ROS for proper cell growth.

A previous study showed that PRDX1 knockout mice developed severe hemolytic anemia and several malignant cancers, including lymphomas, which shortened their life span [10]. The DT40 chicken B-cell line used in the present study is derived from an ALV-induced bursal lymphoma. We speculate that B cell-derived DT40 cells require a lymphoblastic cell-specific cellular environment, which increases susceptibility to the damaging effects of excessive ROS levels.

## Conclusions

In the present study, we demonstrated the critical role of PRDX1 in ROS homeostasis for cell viability. However, the mechanisms underlying the PRDX1-mediated regulation of ROS homeostasis have not yet been elucidated in detail. We found that cell death and the accumulation of ROS in PRDX1-deficient cells were suppressed by Et-SH (Fig. 2). Further investigations are needed to clarify the mechanisms by which cell death and ROS production are suppressed by Et-SH. Our strategy to systematically generate a conditional antioxidant gene mutant in DT40, particularly PRDX1-depleted cells, will become a powerful tool for examining the maintenance of ROS in normal physiological processes.

## Data Availability

All data generated or analyzed during this study are included in this published article.

## Abbreviations

ALV: Avian leukosis virus
APM: L-ascorbic acid phosphate magnesium salt
ATM: Ataxia-telangiectasia mutated
DCFH-DA: 2′,7′-dichlorodihydrofluorescein diacetate
Dox: Doxycycline
Et-SH: 2-mercaptoethanol
ggdPRDX1: *Gallus gallus domesticus* PRDX1
PRDX1: Peroxiredoxin 1
ROS: Reactive oxygen species
RT-PCR: Reverse transcription-PCR
SCE: Sister chromatid exchange
SOD1: Superoxide dismutase 1
SOD2: Superoxide dismutase 2
Tiron: 4,5-dihydroxy-1,3-benzene disulfonic acid
